# The interplay of alcohol use symptoms and sociodemographic factors in the HELIUS study: A network perspective

**DOI:** 10.1007/s00127-025-02954-9

**Published:** 2025-09-01

**Authors:** Karoline B. S. Huth, Orestis Zavlis, Judy Luigjes, Henrike Galenkamp, Anja Lok, Karien Stronks, Claudi L. H. Bockting, Anneke Goudriaan, Maarten Marsman, Ruth J. van Holst

**Affiliations:** 1 Department of Psychology, University of Amsterdam, Amsterdam, the Netherlands; 2 Department of Psychiatry, Amsterdam UMC, University of Amsterdam, Amsterdam, the Netherlands; 3 Centre for Urban Mental Health, University of Amsterdam, Amsterdam, the Netherlands; 4 Department of Public and Occupational Health, University of Amsterdam, Amsterdam, the Netherlands; 5 Health Behaviours and Chronic Diseases, Amsterdam Public Health, Amsterdam, the Netherlands; 6 Arkin Mental Health Institute, Amsterdam, the Netherlands

**Keywords:** HELIUS study, Alcohol use, Network analysis, Sociodemographic factors, Bayesian

## Abstract

**Purpose:**

Research on alcohol use disorder has exclusively focused on either its symptom-level mechanisms–the network perspective or sociodemographic determinants–epidemiological research. Moreover, such research failed to stratify analyses for important person-level factors (e.g., sex or ethnicity). Here, we combine network and epidemiological research and stratify analyses by person-level factors.

**Method:**

Using Bayesian inference, we estimated (1) a logistic regression model predicting past-year alcohol consumption from various sociodemographic factors within a large, multiethnic, urban sample in the Netherlands (complete sample: *N* = 22,164), (2) a cross-sectional network model of alcohol use symptoms and sociodemographic factors among alcohol drinkers of the same sample (drinkers: *N* = 10,877), and (3) stratified networks at the sex- and ethnic- levels in the same drinkers subsample.

**Results:**

All of our examined sociodemographic factors predicted past-year alcohol consumption (in order of magnitude: religion, sex, education, employment, perceived ethnic discrimination, and age). Our Bayesian analysis of networks revealed three notable patterns. First, religion was uniquely and negatively related to adverse alcohol use problems (such as having an injury due to drinking). Second, socioeconomic proxies (education and employment) were positively related to binge drinking, but negatively related to its adverse effects (such as ‘needing a drink in the morning’). Finally, employment and education were particularly negatively related to alcohol use symptoms within male and female networks, respectively.

**Conclusion:**

Our results suggest that alcohol use symptoms are differentially related to sociodemographic factors and that these effects are moderated by sex and ethnicity. Our highlighted network links and Bayesian methodologies could prove useful for future research and prevention and intervention efforts on alcohol use disorders.**Word count**: 4198 words.

**Supplementary Information:**

The online version contains supplementary material available at 10.1007/s00127-025-02954-9.

Alcohol Use Disorder (AUD) is characterized by a pattern of uncontrollable alcohol consumption that persists despite its adverse effects to oneself and others [38]. Increasing evidence points to a model wherein AUD is viewed as a complex system that is generated and maintained through the bidirectional, (non-)linear interactions of various factors, including biological [e.g., genetic effects; 30], psychological [e.g., trait impulsivity; 3], and sociological factors [e.g., socioeconomic status; 25]. Epidemiological research has consistently pointed to the nuanced role of socioeconomic inequalities and ethnic differences on the development of AUD [25]. However, such research has also been limited by its geographical scope (with most studies being conducted in the United States); small and non-representative samples; and the lack of specificity about which AUD symptoms are mostly affected by particular sociodemographic factors [41]. In this study, we address these shortcomings in AUD research by employing Bayesian methods on population-level data from the Netherlands to assess the complex interplay of sociodemographic influences and alcohol use problems.

Prior to our study, most research on AUD has focused on clinical or epidemiological determinants separately. Clinical research using network psychometrics has consistently pointed to the key role of loss of ‘self-control’ within alcohol use networks [31, 45]. Moreover, longitudinal network perspectives have highlighted the different roles that symptoms play in different stages of maladaptive alcohol use. For example, symptoms such as ‘compulsion’ or ‘withdrawal’ tend to be precursors of more advanced AUD symptoms, such as ‘giving up important activities’ [e.g., 45]. These findings are contributing to a history of IRT research assessing the discriminatory role of AUD items, for example, determining tolerance and consuming larger/longer than anticipated to be the least severe items [12]. Combined, the insights supports prominent theories of addiction [e.g., 15] and offer new ways through which they could be extended.

Epidemiological research on AUD has highlighted the crucial role of ethnic, socioeconomic, and religious factors (hereon referred to as sociodemographic factors) in the development of maladaptive alcohol consumption. In Europe, global prevalence rates suggest that ethnic minorities consume less alcohol and exhibit a significantly lower risk for developing AUD, compared to their native counterparts [34, 41]. Nonetheless, ethnic effects on alcohol consumption are conditional on a host of other socio-demographic factors. For example, structural factors such as ethnic or racial discrimination were shown to exacerbate the risk for heavy drinking in ethnic minorities [13, 34]. Moreover, socioeconomic disparities further complicate this dynamic, as they render many minority individuals (particularly recent migrants) with low SES especially vulnerable to the consequences of heavy drinking [19]. Finally, ethnic effects on alcohol consumption are profoundly (and perhaps most robustly) shaped by religiously reinforced cultural identities: While practicing ethno-religious identities (like Muslim Turks or Moroccan Muslims) demonstrate robust protection to alcohol use symptoms due to religious norms, non-religious ethnic minorities like secular Eastern European migrants are at significantly elevated risks for alcohol use difficulties [6, 7, 9]. These patterns underscore the unique role of different socio-cultural factors in structuring European patterns of alcohol consumption in line with postulations of the intersectionality theory [21, 37].

Although most studies acknowledge the effects of sociodemographic factors, few have attempted to examine their unique influences on alcohol use symptoms. Likewise, although the effects of these factors are not uniform across all groups of people [27], recent literature reviews show that researchers rarely stratify their analyses by important grouplevel factors (e.g., ethnic-level or sex-level factors) [28]. Finally, epidemiological research looking into social-level factors has been largely segregated from clinical research, which focuses on symptom-level mechanisms. Integrating the two could help reveal both clinically relevant mechanisms, as well as ways via which they are buffered or aggravated by various sociodemographic determinants.

In this study, we assess how sociodemographic factors are associated with alcohol use symptoms. We address this question within a large, multicultural, European sample, namely, the HELIUS (Healthy Life in an Urban Setting) study from Amsterdam, the Netherlands [32]. We focus on sociodemographic factors that we expect, from previous research, to influence alcohol use symptoms, including perceived ethnic discrimination [13, 34], religion [35], and socioeconomic status [19, 40]. As a first step, we examine the unique effects of these sociodemographic factors on past-year alcohol consumption, similar to past analyses. In a second step, we extend previous analyses, by modeling all effects using network psychometrics, allowing ourselves to investigate the interplay of individual alcohol use symptoms and sociodemographic factors at the population level. Finally, in the last step, we stratify our analyses to assess whether sex or ethnicity moderate the networks of AUD, by affecting the interplay of AUD symptoms and sociodemographic factors. Importantly, we ground our analyses within a Bayesian framework to estimate the uncertainty of our results: that is, how confident we are in the presence or absence of particular connections in our network. Through Bayesian estimation of networks, therefore, we can more clearly distinguish between stable findings and unstable findings.

## Methods

**Sample**

Data were drawn from the Healthy Life in an Urban Setting (HELIUS) study, a largescale and multi-ethnic prospective cohort study conducted in Amsterdam, the Netherlands [20, 32, for more details on the HELIUS study]. Baseline data collection took place from January 2011 to December 2015 [32]. The HELIUS study examined mental and physical health differences among individuals of the largest ethnic groups living in Amsterdam (i.e., Dutch, Surinamese, Ghanaian, Turkish, and Moroccan origin). Of the total 24,789 invited HELIUS participants, we included 23,941 individuals who filled in the questionnaires. From these, we excluded three ethnic groups due to their low sample sizes: the Javanese Surinamese (*N* = 250), the Other/Unknown Surinamese (*N* = 286), and the Other/Unknown ethnicity (*N* = 50). This resulted in a total sample of *N* = 22,164 individuals with an age range of 18 and 73 years. Of the migrants, 75.77% were first-generation migrants and 24.23% second-generation migrants. Importantly, for the network estimation, we only included individuals who consumed alcohol in the past year. Thus, our final network sample included 10,877 participants: *N* = 5487 males (specifically, Dutch *N* = 1946, South-Asian Surinamese *N* = 976, African Surinamese *N* = 1316, Ghanaians *N* = 460, Turks *N* = 575, Moroccans *N* = 214) and *N* = 5390 females (specifically, Dutch *N* = 2158, South-Asian Surinamese *N* = 776, African Surinamese *N* = 1520, Ghanaians *N* = 576, Turks *N* = 276, Moroccans *N* = 93). The sample of drinkers ranged between 18 and 71 years of age and of the migrants that were drinking, 75.40% were first-generation migrants and 24.60% second-generation migrants.

**Measures**

*Alcohol consumption.* Participants indicated whether they consumed alcohol in the past 12 months with a binary ‘yes’ or ‘no’ self-report question.

*Alcohol Use Disorder symptoms.* Alcohol Use Disorder (AUD) symptoms were measured using the Alcohol Use Disorders Identification Test [AUDIT-10; 1]. The AUDIT was developed by the World Health Organization to measure alcohol use problems, through 10 questions with a varying number of categories ranging from three to six [5]. The first question assesses whether participants ‘never drank before’ or ‘had no drinks in the past 12 months’, in which case no further questions are asked. We included the items: binge drinking, unable to stop drinking, unable to engage in normal activities, need drink in the morning, felt guilty due to drinking, unable to retrieve last night, injury due to drinking, and others concerned about drinking.

*Ethnicity.* Following established protocols [11], a participant was deemed as being of non-Dutch ethnic origin if either of the following two conditions were met: (1) the participant was born outside the Netherlands and at least one of their parents was also born outside the Netherlands (i.e., first-generation migrants); (2) the participant was born inside the Netherlands but both of their parents were born outside the Netherlands (i.e., offspring).

*Age and sex.* Age (in years) and sex (male or female) were retrieved from the municipal registry during the data collection period.

***Sociodemographic determinants***

*Employment status.* Employment status was categorized as ‘incapacitated/impaired’, ‘unemployed’, ‘not in the labor force’, and ‘employed’.

*Educational level.* Educational level was measured by self-reported highest educational level in four categories: ‘no or elementary education,’ ‘secondary education,’ intermediate/higher education,’ and ‘higher vocational’.

*Perceived ethnic discrimination.* Perceived Ethnic Discrimination (PED) was captured through the Everyday Discrimination Scale [EDS; 17]. The EDS employs nine items, measured on a five-point Likert scale, ranging from ‘never’ (1) to ‘very often’ (5). The HELIUS study adapted the EDS to reflect discriminatory experiences because of one’s ethnic background [34]. The nine items were aggregated into a sum score.

*Religion.* Religious practices were assessed using a single, binary question measuring whether participants currently ‘practiced a particular religion’.

**Statistical analysis**.

Our code, additional results, and supplementary materials are available at: https://osf.io/jse97/. Our data were analyzed using the statistical software R [49].

***Bayesian predictive modeling***

Using Bayesian logistic regression, we examined the unique effects of our sociodemographic factors on past-year alcohol consumption. Bayesian model averaging was used to account for potential model uncertainty [4], using the BIC criterion as a proxy for the Bayes factor. All sociodemographic variables were considered predictors of past 12 months alcohol use, with equal prior model inclusion probabilities (i.e., prior probablity of 0.5). The posterior model probabilities, inclusion probabilities, and model-averaged means of each predictor were extracted. These analyses were conducted using the BMA package [48].

***Bayesian estimation of symptom networks***

*Data preprocessing.* To accommodate our network analyses, we performed three data pre-processing steps. First, approximately 49% of individuals who responded ‘never had a drink’ or ‘did not drink in the past 12 months’ were excluded from the analysis because they had missing values for all AUD symptoms. As a sensitivity analysis, we repeated our network analyzes with imputed values for these non-drinkers and report these results in our online supplement (see Supplemental Figure S1). Second, the first (frequency of alcohol consumption), second (amount of alcohol consumed), and third (frequency of consuming more than six drinks; binge drinking) items of the AUDIT scale were conceptually similar and highly correlated. To avoid biasing our network structures with highly correlated items [47], we excluded the first two items and included only the latter (i.e., binge drinking) for two reasons: first, it was the most severe indicator of alcohol frequency; second, it was the most similar to other AUD symptoms in our networks (both psychologically in terms of maladaptivity and psychometrically in terms of response scale; see Table S1). Finally, we handled missing values via listwise deletion.

*Bayesian analysis of networks.* Using psychometric network analysis [44, 47], we then examined the interplay among the alcohol use symptoms and our sociodemographic factors; in particular: age, employment, education, religion, and perceived ethnic discrimination. Psychometric networks comprise nodes that represent our variables of interest and edges that represent their associations. In particular, a present edge indicates conditional dependence between variables (i.e., the two variables are associated even after controlling for all other variables in the network), while absent edges indicate conditional independence (i.e., the edges share no association after controlling for all other variables). We conducted a network analysis both at the population level to examine general trends and stratified for sex and ethnicity to examine sex- and ethnic-specific effects.

Notably, we conducted our network analyses within a Bayesian framework to adequately quantify the uncertainty in the networks [46].^1^ Most prominently, the Bayesian approach allows obtaining evidence for the inclusion and exclusion of edges. To accommodate our mixed data (i.e., categorical and continuous variables), we estimated a Gaussian copula Graphical model [GCGM; 14], through the BDgraph R-package [39]. BDgraph implements a Bayesian structure learning approach with a reversible-jump Markov Chain Monte Carlo method [22]. To leverage the Bayesian benefits, one needs to specify their beliefs in the model (e.g., network density) and the parameters (e.g., the strength of network edges) *before* having seen the data: the prior [26, 36]. We adopted the default prior specifications with an edge inclusion probability of 0.5 and three degrees of freedom for the G-Wishart distribution [42].

This analysis yielded three types of undirected networks, visualized using the qgraph package [18]. The first type was the one described previously, in which edges depict the *conditional associations* among our variables. In the second type, edges indicated evidence for *edge presence*.The evidence was quantified using the Bayes Factor. A Bayes factor is a measure used to compare how well two competing explanations or hypotheses explain the observed data, indicating which explanation is more strongly supported by the evidence [2].

Concerning networks, the Bayes factor is used to compare the evidence for edge presence against the evidence for edge absence [46]. For our illustrated networks blue edges indicated strong evidence for edge presence with a Bayes factor greater than 10. In the third network type, the edges indicate evidence for *edge absence*, with red edges indicating a Bayes factor lower than 1/10 which is indicative of strong evidence for edge exclusion. Examining our first network type provides insight into the interplay of sociodemographic factors and alcohol use symptoms. Examining the latter two, however, presents a substantial advancement of previous frequentist network analyses, as it allows us to conclude the evidence for edge presence and importantly edge absence [46].

## Results

Descriptive statistics for the entire study sample(*N* = 22,164), by different ethnic groups, are presented in Table 1. Three notable patterns are evident. First, the group of Dutch origin had the lowest percentage (9%) of alcohol abstinence in the past 12 months, while the groups of Turkish and Moroccan origin reported higher percentages of abstinence (78% and 93%, respectively). Second, a substantial majority of individuals from ethnic minority groups (ranging from 80 to 98%) reported following religious practices, while less than 15% of individuals of Dutch origin reported doing so. Finally, there were substantial differences in education and employment status. Compared to people of Dutch origin, people from ethnic minorities had lower levels of education and were less likely to be employed. Detailed descriptive information on the response to symptoms of alcohol use disorder among drinkers (*N* = 10,877) can be found in our online supplementary Table S1, as well as previous HELIUS publications such as [32] and [41].

**Table 1:** Descriptives

Consume Alcohol	**Dutch** (N = 4509)	**South-Asian Surinamese** (N = 3198)	**African Surinamese** (N = 4179)	**Ghanaian** (N = 2239)	**Turkish** (N = 3860)	**Moroccan** (N = 4179)
	4104 (91%)	1752 (55%)	2836 (68%)	1036 (46%)	842 (22%)	307 (7.3%)
Employment						
Employed	3332 (74%)	1944 (61%)	2615 (63%)	1331 (59%)	2031 (53%)	2030 (49%)
Not in labour force	788 (17%)	467 (15%)	497 (12%)	179 (8.0%)	912 (24%)	1156 (28%)
Unemployed	252 (5.6%)	492 (15%)	680 (16%)	532 (24%)	571 (15%)	652 (16%)
Incapacitated	137 (3.0%)	295 (9.2%)	387 (9.3%)	197 (8.8%)	346 (9.0%)	341 (8.2%)
Education						
No/Elementary	149 (3.3%)	439 (14%)	228 (5.5%)	615 (27%)	1196 (31%)	1256 (30%)
Secondary	640 (14%)	1056 (33%)	1499 (36%)	903 (40%)	967 (25%)	759 (18%)
Higher Secondary	983 (22%)	975 (30%)	1517 (36%)	576 (26%)	1133 (29%)	1432 (34%)
Higher Vocational	2737 (61%)	728 (23%)	935 (22%)	145 (6.5%)	564 (15%)	732 (18%)
Religion						
Non-Religious	3918 (87%)	635 (20%)	952 (23%)	244 (11%)	251 (6.5%)	77 (1.8%)
Religious	591 (13%)	2563 (80%)	3227 (77%)	1995 (89%)	3609 (93%)	4102 (98%)
Perc. Ethnic Discr.	10 (3)	18 (7)	18 (7)	17 (7)	17 (7)	18 (7)
Age	46 (14)	45 (14)	47 (13)	44 (12)	40 (12)	40 (13)
Sex						
Male	2078 (46%)	1480 (46%)	1685 (40%)	880 (39%)	1722 (45%)	1579 (38%)
Female	2431 (54%)	1718 (54%)	2494 (60%)	1359 (61%)	2138 (55%)	2600 (62%)

Note. For continuous variables (i.e., perceived ethnic discrimination and age), the mean and standard deviation are shown; for all other variables, the respective count and percentage are shown

**Aim 1: The prediction of past-year alcohol consumption**

In the logistic regression, all sociodemographic variables showed a posterior inclusion probability of one (i.e., *p*_*inc*_ = 1). The results favor the model that includes all variables to predict past-year alcohol consumption in the sample of drinkers and non-drinkers. Religion (*β* = *−*2.04, *β*_*se*_ = 0.04), perceived ethnic discrimination (*β* = *−*0.07, *β*_*se*_ = 0.02) and sex (*β* = *−*0.62, *β*_*se*_ = 0.03) negatively predicted alcohol consumption (see Table 2). Religious individuals showed a lower chance of consuming alcohol than did the non-religious ones (Odds Ratio: 10.98), similar to female individuals over males (Odds Ratio: 1.90). In contrast, age (*β* = 0.023, *β*_*se*_ = 0.02), employment (*β* = 0.11, *β*_*se*_ = 0.02), and education (*β* = 0.45, *β*_*se*_ = 0.02) positively predicted alcohol consumption. Older and educated individuals were more likely to consume alcohol in the past year.

**Aim 2: The interplay of alcohol use symptoms and sociodemographic factors at the population level**

**Fig. 1:**
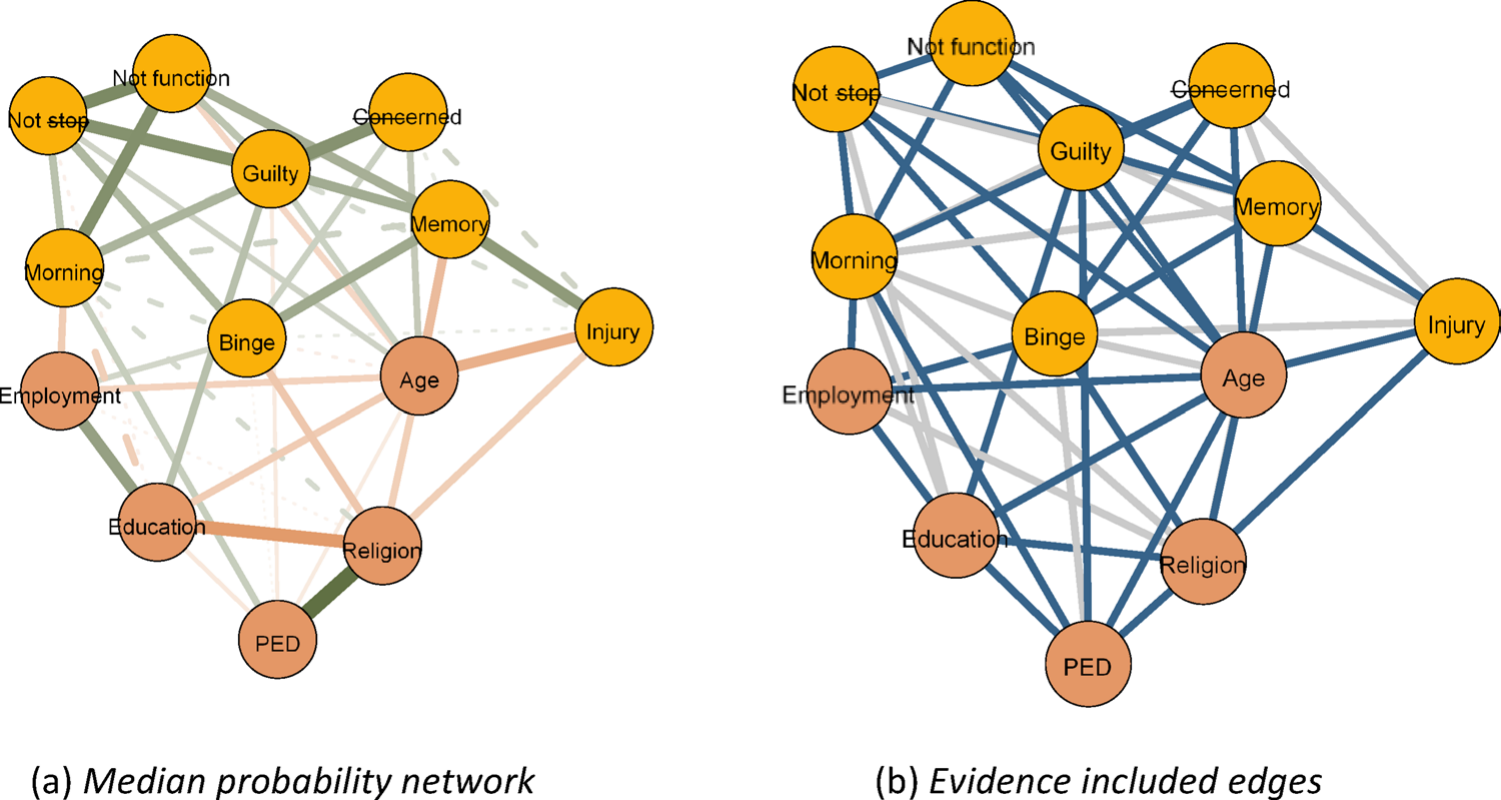
Network of AUD symptoms and sociodemographic factors for all participants. **a** Median probability network, **b** evidence included edges. *Note*. In the *left panel*, edge thickness and saturation indicate the strength of the association, with orange representing negative relationships and green representing positive relationships. Dashed lines represent edges with inconclusive evidence of presence ($1 < BF_{inc} < 10$). In the *right panel*, blue edges indicate evidence for inclusion ($BF_{inc} > 10$) and gray edges indicate no evidence for inclusion or exclusion ($1 < BF_{inc} < 10$). *Nodes*: Binge—binge drinking, Not stop—unable to stop drinking, Not function—unable to do normal activities, Morning—need a drink in the morning, Guilty—felt guilty due to drinking, Memory—unable to retrieve last night, Injury—injury due to drinking, Concerned—others concerned, PED—perceived ethnic discrimination

Figure 1 displays the interplay between AUD symptoms and sociodemographic factors at the population level as well as the evidence supporting included edges (see Figure S2 for the evidence for absent edges). Extending the previous logistic analyses, this populationlevel network highlights at least two interesting patterns. First, sociodemographic factors map differentially on AUD symptoms. For example, while age exhibited negative associations with some AUD symptoms like (1) drinking-related injury (*θ*_*ij*_ = − 0.22, *BF*_*inc*_*>*3000) and (2) unable to function due to drinking(*θ*_*ij*_ = −.11, *BF*_*inc*_*>* 3000), it also exhibited some positive associations to other AUD symptoms, like (1) inability to stop drinking (*θ*_*ij*_ =.10, *BF*_*inc*_ = 15.6), (2) feeling guilty due to drinking (*θ*_*ij*_ =.11, *BF*_*inc*_*>* 3000), and (3) others being concerned (*θ*_*ij*_ =.13, *BF*_*inc*_*>* 3000). Likewise, while perceived ethnic discrimination was negatively and weakly associated with less severe symptoms like ‘feeling guilty due to drinking’ (*θ*_*ij*_ = − 0.06, *BF*_*inc*_ = 10.1), it was positively and strongly associated with more severe functional symptoms like ‘needing a drink in the morning’ (*θ*_*ij*_ = 0.12, *BF*_*inc*_ = 15.6). Notably, a similar pattern was found in sociodemographic factors: While both employment and education were positively related to some AUD symptoms, like binge drinking (*θ*_*ij*_ = 0.09, *BF*_*inc*_*>* 3000) and ‘feeling guilty due to drinking’ (*θ*_*ij*_ = 0.15, *BF*_*inc*_*>* 3000), respectively, they were also negatively related to more severe AUD symptoms, like ‘needing a drink in the morning’ which was most conclusively related to employment (*θ*_*ij*_ = 0.14, *BF*_*inc*_ = 9). Finally, one predictor (religion) exhibited only negative associations, specifically, with severe AUD symptoms like (1) binge drinking (*θ*_*ij*_ = − 0.15, *BF*_*inc*_*>* 3000), (2) having no memory of a drinking night (*θ*_*ij*_ = − 0.03, *BF*_*inc*_ = 1.12), and (3) injury due to drinking (*θ*_*ij*_ = − 0.13, *BF*_*inc*_*>*3000).

A second pattern from our population-level network includes the observation that the sociodemographic factors were highly correlated among themselves. Specifically, perceived ethnic discrimination was strongly associated with religion (*θ*_*ij*_ =.35, *BF*_*inc*_*>* 3000), which in turn was negatively related to education (*θ*_*ij*_ = −.28, *BF*_*inc*_*>* 3000) which finally mapped positively on employment (*θ*_*ij*_ =.23, *BF*_*inc*_*>* 3000). A similar pattern was evident for symptom-specific patterns, with severe indicators of alcohol use being positively inter-related, e.g., ‘not functioning’ being strongly related to both ‘unable to stop’ (*θ*_*ij*_ =.29, *BF*_*inc*_*>* 3000) and ‘needing a drink in the morning’ (*θ*_*ij*_ = 0.27, *BF*_*inc*_*>*3000). These patterns suggest that there might exist specific clustering effects, although we note that such effects cannot be inferred from our present analyses.

**Table 2:** Results of the logistic regression

Variables	PIP	βmean	βsd
Intercept	1.00	0.31	0.08
Employment	1.00	0.11	0.02
Education	1.00	0.45	0.02
Religion	1.00	−2.04	0.04
Percei. Ethnic Disc.	1.00	−0.07	0.02
Age	1.00	0.23	0.02
Sex	1.00	−0.62	0.03

Note. PIP = Posterior inclusion probability; SD = standard deviation

**Aim 3: The moderating effects of sex and ethnicity on the interplay of alcohol use symptoms and sociodemographic factors**

**Fig. 2:**
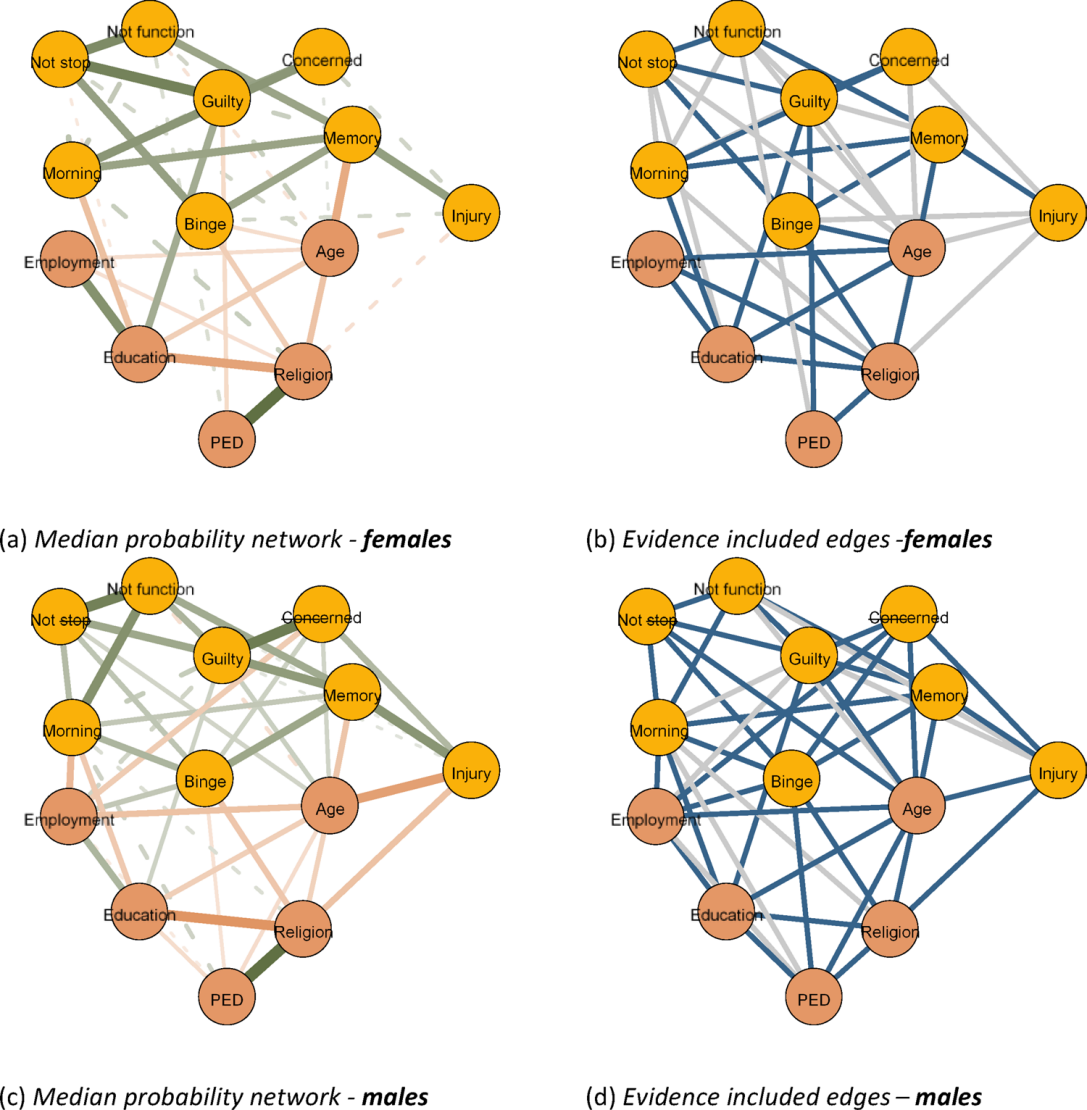
Network of alcohol use disorder symptoms and sociodemographic determinants **split by sex**. **a** Median probability network - females, **b** evidence included edges - females, **c** median probability network - males and **d** evidence included edges - males. *Note*. The networks show interactions between AUD symptoms and sociodemographic determinants for females (top row) and males (bottom row). In the *left column*, edge thickness and saturation indicate the strength of the association, with orange representing negative relationships and green representing positive relationships. Dashed lines represent edges with inconclusive evidence of presence (1 < *BF*_*inc*_ < 10). In the *right column*, blue edges indicate evidence for inclusion (*BF*_*inc*_ > 10) and gray edges indicate no evidence for inclusion or exclusion (1 < *BF*_*inc*_ < 10). *Nodes*: Binge—binge drinking, Not stop—unable to stop drinking, Not function—unable to do normal activities, Morning—need a drink in the morning, Guilty—felt guilty due to drinking, Memory—unable to retrieve last night, Injury—injury due to drinking, Concerned—others concerned, PED—perceived ethnic discrimination

*Sex-specific Networks.* Figure 2  displays the sex-specific networks of AUD symptoms and sociodemographic factors among drinkers(see Figure S3 for the evidence plotso). Overall, three patterns are notable. First, the associations among AUD symptoms are strongly and exclusively positive. Notable associations that remain invariant across the sex-specific networks include (1) ‘felt guilty’, which is strongly linked with ‘unable to stop drinking’ (females: θ_ij_=.33, *BF*_*inc*_*>*3000; males: *θ*_*ij*_ =.21, *BF*_*inc*_*>*3000) and ‘others concerned’ (females: *θ*_*ij*_ = 0.25, *BF*_*inc*_= 11.5; males: *θ*_*ij*_ = 0.31, *BF*_*inc*_*> *3000) and (2) being ‘unable to stop drinking’, which is strongly linked with being ‘unable to do normal activities’ (females: *θ*_*ij*_ = 0.31, *BF*_*inc*_*> *3000; males: *θ*_*ij*_ = 0.30, *BF*_*inc*_*>* 3000). Only the edge between ‘unable to do normal activities’ and ‘others concerned’ in the male network shows evidence for conditional independence between symptoms (*θ*_*ij*_= 0.002, *BF*_*inc*_ = 0.06); all other AUD symptom interconnections show evidence for the inclusion of the edge or inconclusive evidence.

Second, extending our population-level analyses, some associations between our sociodemographic predictors and AUD symptoms here show nuanced patterns. For instance, *PED *is negatively related to ‘binge drinking’ (*θ*_*ij*_= − 0.08, *BF*_*inc*_ = 32.33) in males. Similarly, in females, *PED* is negatively related to ‘feeling guilty about drinking’ (*θ*_*ij*_ = − 0.11, *BF*_*inc*_*>*3000). In men, age is positively related to ‘feeling guilty about drinking’ (*θ*_*ij*_ = 0.11, *BF*_*inc*_*>*3000).

Finally, two key differences emerged across our sex-specific networks. First, although *employment* shows no relationship with symptoms in females, it is highly interconnected with symptoms in males. In particular, in males, employment is strongly and negatively related to ‘needing a drink in the morning’ ($\theta_{ij} = -0.19$, $BF_{inc} > 3000$) and ‘others concerned’ ($\theta_{ij} = -0.14$, $BF_{inc} = 99.00$), as well as weakly (both in magnitude and evidence for presence) and positively associated with ‘feeling guilty due to drinking’ ($\theta_{ij} =.10$, $BF_{inc} = 9.00$). A second, converse pattern includes *education*, which is more strongly interconnected with symptoms in females than males. In particular, education is negatively related to ‘needing a drink in the morning’ (females: $\theta_{ij} = -0.20$, $BF_{inc} = 19.00$; males: $\theta_{ij} = -0.14$, $BF_{inc} > 3000$) and positively related to ‘feeling guilty’ (females: $\theta_{ij} =.22$, $BF_{inc} > 3000$; males: $\theta_{ij} =.11$, $BF_{inc} > 3000$), as well as weakly (in both magnitude and presence) and negatively related to ‘unable to stop drinking’, only in females ($\theta_{ij} = -0.07$, $BF_{inc} = 1.38$).

**Fig. 3:**
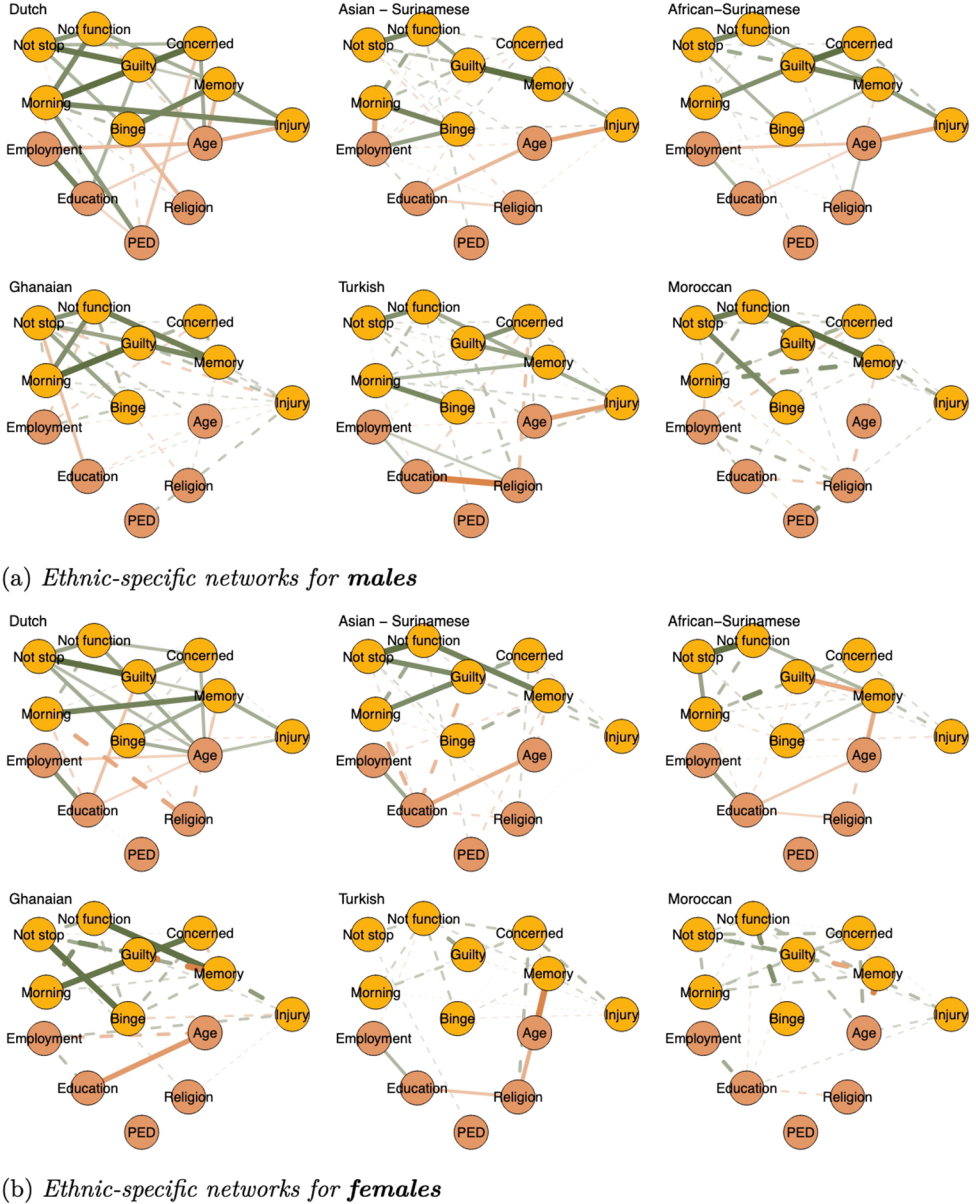
Sex-specific network plots of alcohol use disorder symptoms and sociodemographic determinants differentiated by ethnic groups. **a** Ethnic-specific networks for **males**, **b** ethnic-specific networks for **females**. *Note.* Edge thickness and saturation represent the strength of the association; the thicker the edge, the stronger the association. Orange edges indicate negative relations and green edges positive ones. Dashed lines indicate edges with inconclusive evidence for presence (i.e., 1 < *BF*_*inc*_ < 10). *Nodes*: Binge—binge drinking, Not stop–unable to stop drinking, Not function—unable to do normal activities, Morning—need a drink in the morning, Guilty—felt guilty due to drinking, Memory—unable to retrieve last night, Injury—injury due to drinking, Concerned—others concerned, PED—perceived ethnic discrimination

*Ethnicity-specific Networks.* Figure 3 shows the ethnic-specific networks for males and females. In each subgroup, sociodemographic determinants are differentially associated with AUD symptoms. Some subgroups show minimal interactions between AUD symptoms and sociodemographic determinants (e.g. African-Surinamese, Moroccan, and Ghanaian women). Others show similar dependence on all sociodemographic determinants, including Turkish males, Ghanaian males, and Dutch individuals. In addition, certain subgroups show strong associations with one particular socio-demographic determinant and weaker associations with others. For example, Asian-Surinamese males show strong associations with employment and Asian-Surinamese females with education. Similarly, Asian-Surinamese and Turkish females exhibit positive relations between perceived ethnic discrimination and a particular functional symptom (’inability to do normal activities). However, the edges in the subgroup networks show mostly inconclusive evidence, limiting further description of symptom-specific patterns (see the edge evidence plots by ethnic groups for males and females respectively in the supplemental Figures S4 and S5). We also ran a ethnic specific network model for both males and females combined to increase the sample size per model (see supplemental Figure S6 for the network plots and S7 for the edge evidence plots). The increased sample sizes did make some edges more certain but did not substantially increase the confidence that can be placed in the network.

## Discussion

To our knowledge, this is the first study to use network psychometrics, in general, and Bayesian methods, in particular, to examine the complex interplay of alcohol use symptoms and sociodemographic factors in a large, representative sample of the Dutch population. Using common regression models, we replicated previous research by showcasing that various sociodemographic factors (most notably, religion) are important, unique predictors of past-year alcohol consumption. Using more novel methodologies such as network psychometrics, we revealed that our sociodemographic factors differentially affect individual alcohol use symptoms. Finally, by stratifying our network analyses, we highlighted that these effects were moderated by sex and ethnicity, stressing the importance of accounting for such demographic factors when examining alcohol use symptoms. Below, we outline our findings in detail, focusing on both their theoretical and methodological contributions.

First, practicing a religion reduced the odds of consuming alcohol in the past year in line with existing research [35]. Extending previous research, though, our populationlevel network revealed that religion was specifically associated with several alcohol use symptoms: ‘binge drinking’, ‘having no memory of a drinking night’, and ‘injury due to (i.e.,1*<* BF_inc_*<*10).*Nodes:* drinking’. These patterns suggest that religious beliefs may specifically affect these particular alcohol symptoms, by decreasing the probability of binge drinking and buffering against some of its adverse consequences, for example, alcohol-related injuries. Interestingly, similar patterns emerged across all ethnic-specific networks, suggesting that these effects may remain invariant across different religions. Indeed, in keeping with this interpretation, ample evidence suggests that spirituality more broadly is an effective intervention/prevention strategy against various addiction problems [8, 10, 31].

Second, a higher socioeconomic status increased the odds of past-year alcohol consumption, replicating previous findings [19, 27, 40]. Although this pattern could suggest that people of higher socioeconomic status are at a higher risk for developing alcohol-related problems, when looking at the symptom-level patterns a more nuanced picture emerges. In particular, our two socioeconomic indicators (education and employment) exhibited both positive and negative relations with alcohol use symptoms. For instance, both education and employment were negatively related to certain alcohol dependence symptoms (such as ‘needing a drink in the morning’), yet also positively related with ‘binge drinking’ and ‘feeling guilty due to drinking.’ These patterns suggest that people of higher socioeconomic status could have a reduced likelihood of developing alcohol dependence symptoms, despite having a higher likelihood of drinking. These patterns further stress the importance of more granular, symptom-level analyses as a means of specifying general, disorder-level findings.

A third, related pattern concerns sex-specific differences in alcohol use dynamics. Specifically, the most important negative predictors of alcohol use symptoms were education for females and employment for males. These differential associations suggest that socioeconomic factors may play distinct roles in shaping alcohol-related behaviors across sexes. For instance, the protective role of education in females may stem from higher education granting women greater economic autonomy, social capital, and access to healthcare, which may buffer against stressors linked to alcohol use [24, 25, 33]. Conversely, the protective role of employment in males could be tied to cultural stereotypes that equate male identity to labor participation: in such contexts, unemployment may exacerbate feelings of inadequacy and a loss of purpose, driving coping behaviors like excessive alcohol consumption [16, 23]. Although these patterns make sense in light of this previous research, we note that our interpretation of them remains exploratory and speculative given that our study is cross-sectional and does not examine how broader systemic variables such as structural inequalities and sex-specific stereotypes mediate our observed associations. Accordingly, a fruitful direction for future research could be to integrate these individual-level data with macro-level data, examining how cultural forces interact with education and employment to influence alcohol consumption in different gender groups.

Finally, we note some intriguing results relating to ethnic-specific effects, particularly regarding ethnic discrimination. In our population-network, ethnic discrimination was strongly associated with religion, an effect likely reflecting the strong intersection of religion with certain ethnicities. Notably, religion was also negatively associated with education, suggesting that it may diminish the protective effects of education in particularly religious minority populations. Consistent with this interpretation, perceived ethnic discrimination was found to be associated with functional AUD symptoms (like ‘inability to do normal activities’) but only within females of specific ethnicities (like Asian-Surinamese and Turkish). These patterns point to novel, ethnicity-specific effects, illustrating the importance of stratifying statistical analyses by both sex and ethnicity. However, although interesting, our interpretation of these effects remains limited because their networks were highly unstable.

This brings us to the strengths and limitations of our investigation. To elaborate, our study has some notable strengths, including its large, representative sample; use of novel Bayesian network methods; and stratification of statistical analyses according to sex and ethnicity. Nevertheless, some limitations must be acknowledged. First, our findings may only be applicable to the specific population of Amsterdam, the Netherlands. Likewise, our statistical trends may not be generalizable to clinical populations, given previous research showcasing differences in alcohol use networks across clinical and nonclinical populations [e.g., 43]. Second, it is important to note that our study is cross-sectional, so our observed effects should be interpreted as associations rather than causal connections. For instance, while unemployment may be associated with increased alcohol use, it could also be a consequence of problematic alcohol use and absenteeism. To unpack the causality of these effects (both between and within participants) longitudinal network perspectives will be necessary. Third, we note that some of our ethnic-specific effects were likely inflated because our networks were constructed using populations who consumed alcohol. Given that for many ethnicities consuming alcohol remains a culturally and religiously prohibited activity, the people who reported consuming alcohol had likely many other inflated symptoms. Future work may wish to conduct more ethnic-specific examinations of alcohol consumption, including both drinkers and non-drinkers to identify more specific risk factors. Finally, it should be noted that the data was collected more than ten years ago and as such some of the findings might be altered or differently interpreted in the changing cultural norms.

## Conclusion

To conclude, our study sheds considerable light on the complex interplay of alcohol use symptoms and sociodemographic factors. Replicating previous research, we showed that various sociodemographic factors were important, unique predictors of past-year alcohol use. Extending previous research, we highlighted that religion was particularly protective of adverse alcohol use problems; education buffered against alcohol use problems for females; and employment played the corresponding role for males. Although our ethnic-specific networks were somewhat unstable and limited inductive inferences, they nonetheless highlighted the differential effects of perceived ethnic discrimination across ethnic minorities. Our findings stress the importance of considering sociodemographic factors in research as well as clinical settings and encourage the use of Bayesian methods as a means of further estimate the uncertainty underlying ones findings and thereby the confidence that can be placed in reporting the networks.

## Electronic supplementary material

Below is the link to the electronic supplementary material.

Supplementary Material 1

## Data Availability

The data that support the findings of this study are available from HELIUS, but restrictions apply to the availability of these data, which were used under licence for the current study and so are not publicly available. The data are, however, available from the HELIUS initiative following their data access policy https://www.heliusstudy.nl/en/researchers/.

## Footnotes

1 A detailed description of the Bayesian analysis of networks and its advantage is beyond the scope of this paper. Interested readers are referred to [46].
