# Supplementary material for: The interplay of alcohol use symptoms and sociodemographic factors in the HELIUS study: A network perspective
Source: Soc Psychiatry Psychiatr Epidemiol. 2025 Sep 1;60(11):2577–87. doi: 10.1007/s00127-025-02954-9 (PMC12572036; doi:10.1007/s00127-025-02954-9)
Supplement: Supplementary file 1 — Supplementary Material 1 [file 127_2025_2954_MOESM1_ESM.pdf]

SUPPLEMENTAL APPENDIX: The interplay of alcohol use symptoms and sociodemographic factors in the HELIUS study: A network perspective

Huth, K.B.S.*[1,2,3], Zavlis, O.*[2], Luigjes, J.[2,3], Galenkamp, H.[4,5], Lok, A.[2,3,5], Stronks, K.[3,4,5], Bockting, C.L.H.[2,3], Goudriaan, A.E. [2,3,6], Marsman, M.[1,3], & van Holst, R.J. [2,3]

1 Department of Psychology, University of Amsterdam

2 Department of Psychiatry, Amsterdam UMC, University of Amsterdam

3 Centre for Urban Mental Health, University of Amsterdam

4 Department of Public and Occupational Health, Amsterdam UMC, University of Amsterdam

5 Amsterdam Public Health, Health Behaviours and Chronic Diseases, Amsterdam

6 Arkin Mental Health Institute, the Netherlands

∗ Indicates shared first authorship

Abstract

Purpose: Research on alcohol use disorder has exclusively focused on either its symptom-level mechanisms–the network perspective– or sociodemographic determinants–epidemiological research. Moreover, such research failed to stratify analyses for important person-level factors (e.g., sex or ethnicity). Here, we combine network and epidemiological research and stratify analyses by person-level factors.
Method: Using Bayesian inference, we estimated (1) a logistic regression model predicting past-year alcohol consumption from various sociodemographic factors within a large, multiethnic, urban sample in the Netherlands (complete sample: $N = 22,164$), (2) a cross-sectional network model of alcohol use symptoms and sociodemographic factors among alcohol drinkers of the same sample (drinkers: $N = 10,877$), and (3) stratified networks at the sex- and ethnic- levels in the same drinkers subsample.
Results: All of our examined sociodemographic factors predicted past-year alcohol consumption (in order of magnitude: religion, sex, education, employment, perceived ethnic discrimination, and age). Our Bayesian analysis of networks revealed three notable patterns. First, religion was uniquely and negatively related to adverse alcohol use problems (such as having an injury due to drinking). Second, socioeconomic proxies (education and employment) were positively related to binge drinking, but negatively related to its adverse effects (such as 'needing a drink in the morning'). Finally, employment and education were particularly negatively related to alcohol use symptoms within male and female networks, respectively.
Conclusion: Our results suggest that alcohol use symptoms are differentially related to sociodemographic factors and that these effects are moderated by sex and ethnicity. Our highlighted network links and Bayesian methodologies could prove useful for future research and clinical work on alcohol use disorders.

Keywords: HELIUS study, alcohol use, network analysis, sociodemographic, Bayesian

Table S1

Descriptives of alcohol use disorder variables of individuals that consumed in the past year by sex.

	**Male** N = 5,487	**Female** N = 5,390
Binge Drinking		
Never	2,094 (38%)	3,403 (63%)
Less than once a month	1,594 (29%)	1,229 (23%)
Once a month	955 (17%)	502 (9.3%)
Once a week	689 (13%)	231 (4.3%)
(Almost) everyday	155 (2.8%)	25 (0.5%)
Unable to stop		
Never	4,631 (84%)	4,917 (91%)
Less than once a month	449 (8.2%)	288 (5.3%)
Once a month	192 (3.5%)	89 (1.7%)
Once a week	142 (2.6%)	68 (1.3%)
(Almost) everyday	73 (1.3%)	28 (0.5%)
Unable to do normal activities		
Never	4,880 (89%)	5,080 (94%)
Less than once a month	425 (7.7%)	233 (4.3%)
Once a month	97 (1.8%)	49 (0.9%)
Once a week	52 (0.9%)	15 (0.3%)
(Almost) everyday	33 (0.6%)	13 (0.2%)
Need drink in the morning		
Never	5,323 (97%)	5,336 (99%)
Less than once a month	78 (1.4%)	35 (0.6%)
Once a month	31 (0.6%)	7 (0.1%)
Once a week	29 (0.5%)	8 (0.1%)
(Almost) everyday	26 (0.5%)	4 (<0.1%)
Felt guilty due to drinking		
Never	4,420 (81%)	4,650 (86%)
Less than once a month	659 (12%)	521 (9.7%)
Once a month	192 (3.5%)	132 (2.4%)
Once a week	117 (2.1%)	54 (1.0%)
(Almost) everyday	99 (1.8%)	33 (0.6%)
Unable to retrieve last night		
Never	4,733 (86%)	4,959 (92%)
Less than once a month	565 (10%)	357 (6.6%)
Once a month	123 (2.2%)	51 (0.9%)
Once a week	39 (0.7%)	12 (0.2%)
(Almost) everyday	27 (0.5%)	11 (0.2%)
Injury due to drinking		
Never	5,131 (94%)	5,231 (97%)
Not in past year	283 (5.2%)	119 (2.2%)
Yes, in past year	73 (1.3%)	40 (0.7%)
Others concerned		
Never	4,828 (88%)	5,158 (96%)
Not in past year	297 (5.4%)	108 (2.0%)
Yes, in past year	362 (6.6%)	124 (2.3%)

Figure S1

Networks based on ***non-drinkers and drinkers combined****.*

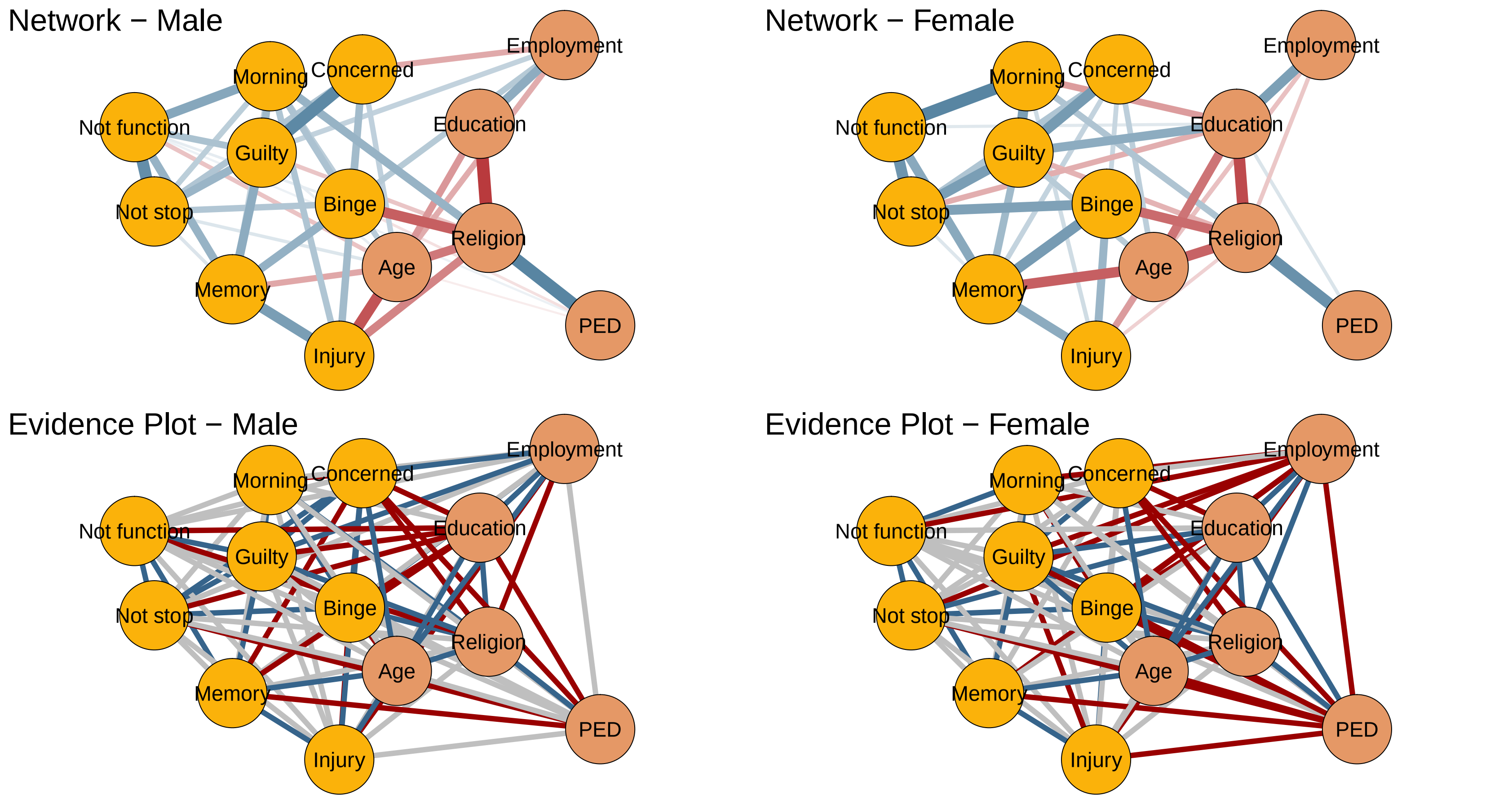

Note. Networks depicting the partial associations between AUD symptoms and sociodemographic determinants (top row) and the respective network uncertainty (bottom row) for females (right column) and males (left column). *Top row:* Edge thickness and saturation represent the strength of association, the thicker the edge, the stronger the association. Red edges indicate negative relations and blue edges, positive ones. *Bottom row:* In the edge evidence plots, edges represent evidence for inclusion, where blue edges represent evidence for inclusion ($BF_{inc} >$ 10), red edges evidence for exclusion ($BF_{inc} < 0.1$), and grey edges absence of evidence for either in-or exclusion ($0.1 < BF_{inc} < 10$). *Nodes:* Binge–binge drinking, Not stop–unable to stop drinking, Not function–unable to do normal activities, Morning–need a drink in the morning, Guilty–felt guilty due to drinking, Memory–unable to retrieve last night, Injury–injury due to drinking, Concerned–others concerned, PED–perceived ethnic discrimination.

Figure S2

Evidence plot for absent edges - all participants.

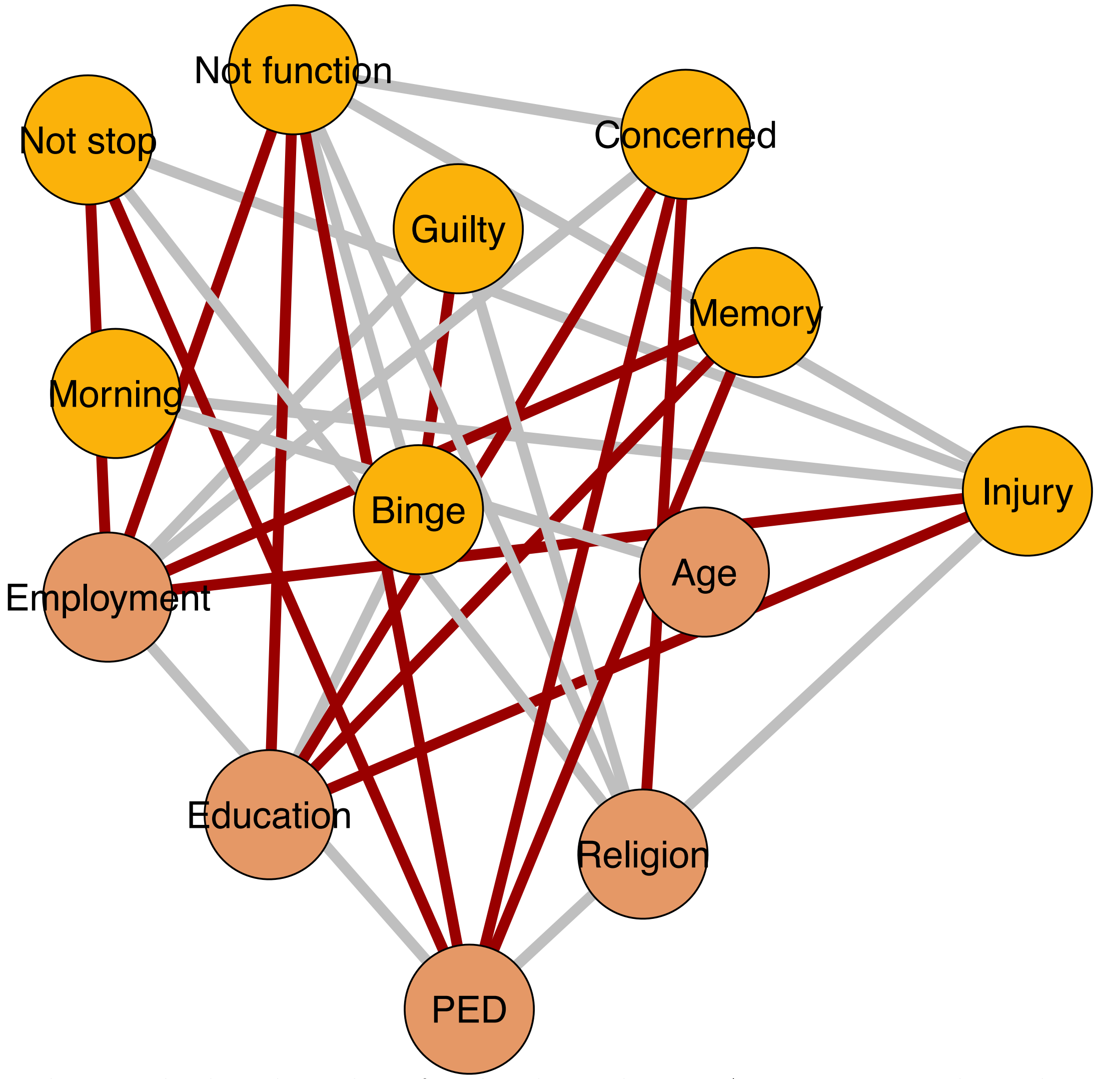

Note. The networks show the evidence for edge absence between AUD symptoms and sociodemographic determinants for all participants. The edge evidence plot show inclusion Bayes factors for all edges with some evidence for exclusion ($BF_{inc} < 1$). Red edges indicate evidence for exclusion ($BF_{inc} < 0.1$) and gray edges indicate some but not conclusive evidence for edge exclusion ($0.1 < BF_{inc} < 1$). *Nodes:* Binge–binge drinking, Not stop–unable to stop drinking, Not function–unable to do normal activities, Morning–need a drink in the morning, Guilty–felt guilty due to drinking, Memory–unable to retrieve last night, Injury–injury due to drinking, Concerned–others concerned, PED–perceived ethnic discrimination.

Figure S3

Evidence plot for absent edges - split by sex

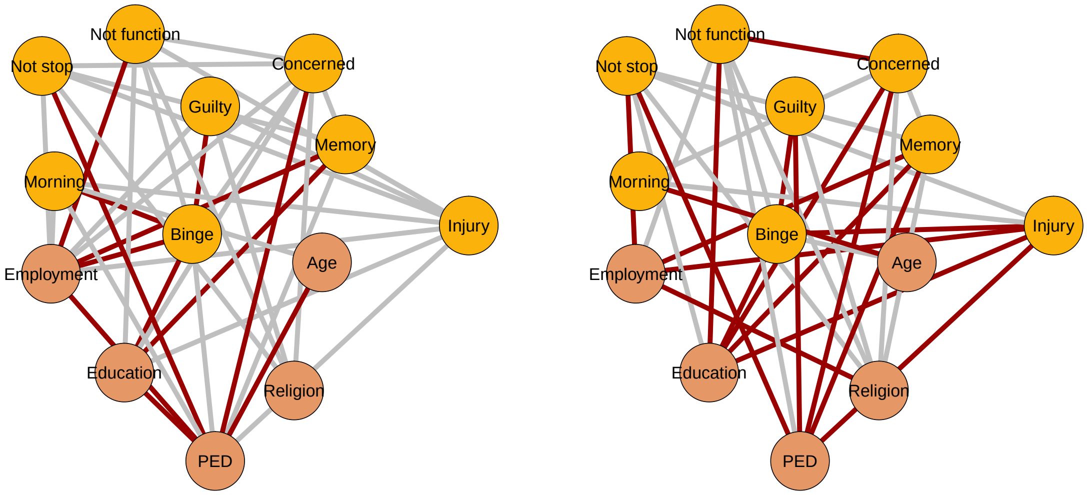

(a) *Evidence excluded edges - females* (b) *Evidence excluded edges - males*

Note. The networks show the evidence for edge absence between AUD symptoms and sociodemographic determinants for females (left) and males (right). The edge evidence plot show inclusion Bayes factors for all edges with some evidence for exclusion ($BF_{inc} < 1$). Red edges indicate evidence for exclusion ($BF_{inc} < 0.1$) and gray edges indicate some but not conclusive evidence for edge exclusion ($0.1 < BF_{inc} < 1$). *Nodes:* Binge–binge drinking, Not stop–unable to stop drinking, Not function–unable to do normal activities, Morning–need a drink in the morning, Guilty–felt guilty due to drinking, Memory–unable to retrieve last night, Injury–injury due to drinking, Concerned–others concerned, PED–perceived ethnic discrimination.

Figure S4

Edge evidence plots for ***males*** *of the various ethnic subgroups.*

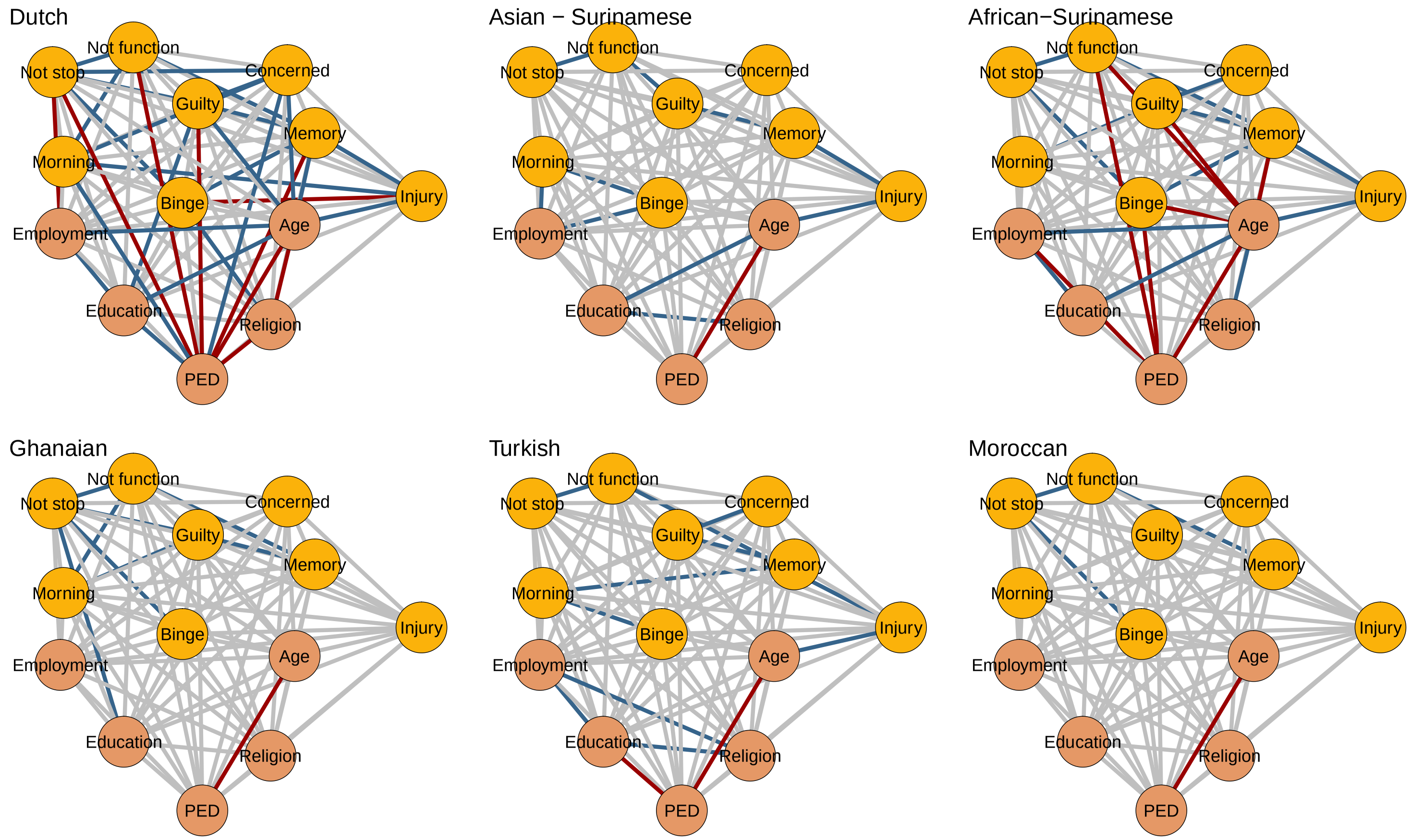

Note. Networks depicting the respective network uncertainty for males in the ethnic subgroups. In the edge evidence plots, edges represent evidence for inclusion, where blue edges represent evidence for inclusion ($BF_{inc} > 10$), red edges evidence for exclusion ($BF_{inc} < 0.1$), and grey edges absence of evidence for either in- or exclusion ($0.1 < BF_{inc} < 10$). *Nodes:* Binge–binge drinking, Not stop–unable to stop drinking, Not function–unable to do normal activities, Morning–need a drink in the morning, Guilty–felt guilty due to drinking, Memory–unable to retrieve last night, Injury–injury due to drinking, Concerned–others concerned, PED–perceived ethnic discrimination.

Figure S5

*Edge evidence plots for **females** of the various ethnic subgroups.*

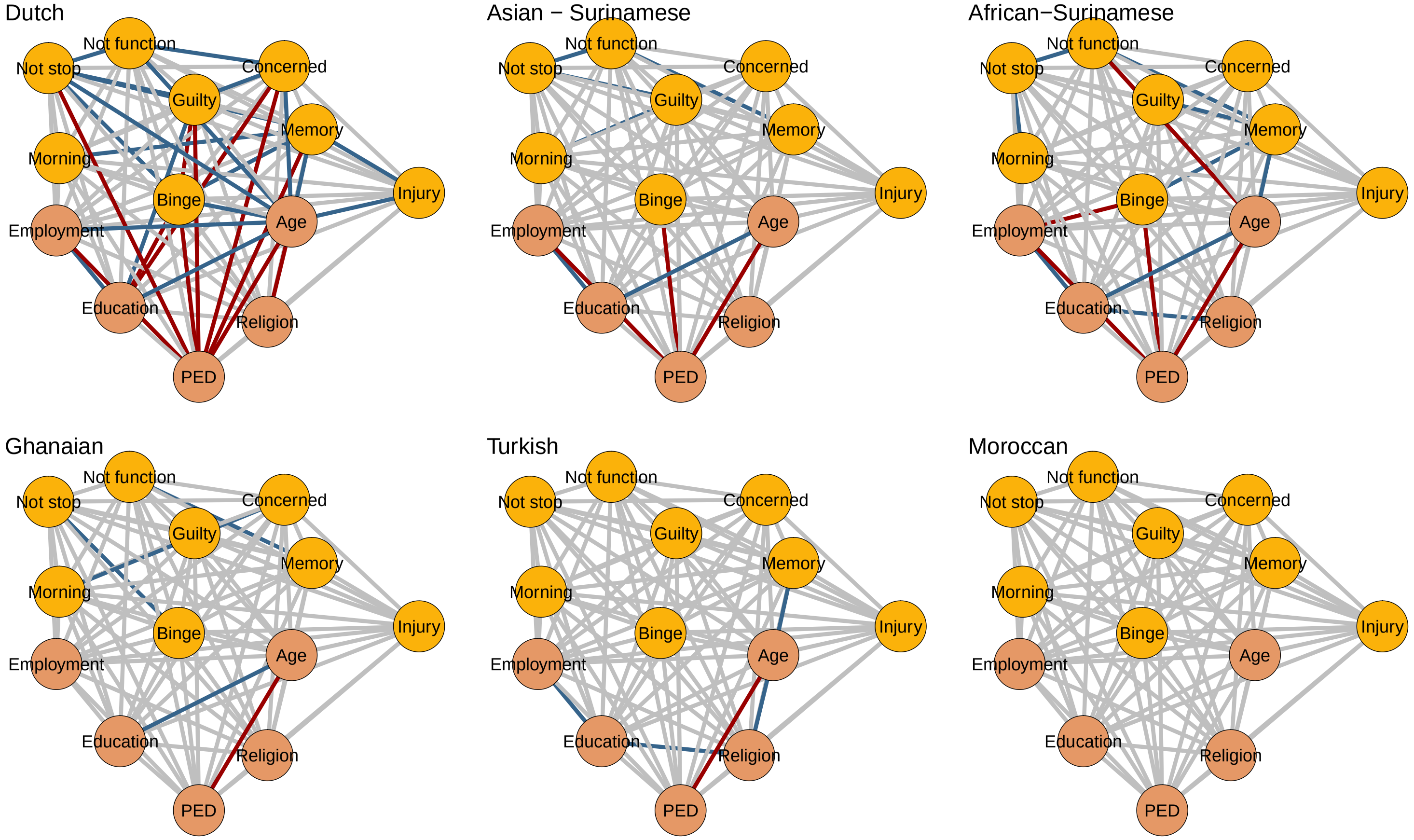

Note. Networks depicting the respective network uncertainty for females in the ethnic subgroups. In the edge evidence plots, edges represent evidence for inclusion, where blue edges represent evidence for inclusion ($BF_{inc} > 10$), red edges evidence for exclusion ($BF_{inc} < 0.1$), and grey edges absence of evidence for either in- or exclusion ($0.1 < BF_{inc} < 10$). *Nodes:* Binge–binge drinking, Not stop–unable to stop drinking, Not function–unable to do normal activities, Morning–need a drink in the morning, Guilty–felt guilty due to drinking, Memory–unable to retrieve last night, Injury–injury due to drinking, Concerned–others concerned, PED–perceived ethnic discrimination.

Figure S6

Networks for both sexes of the various ethnic subgroups.

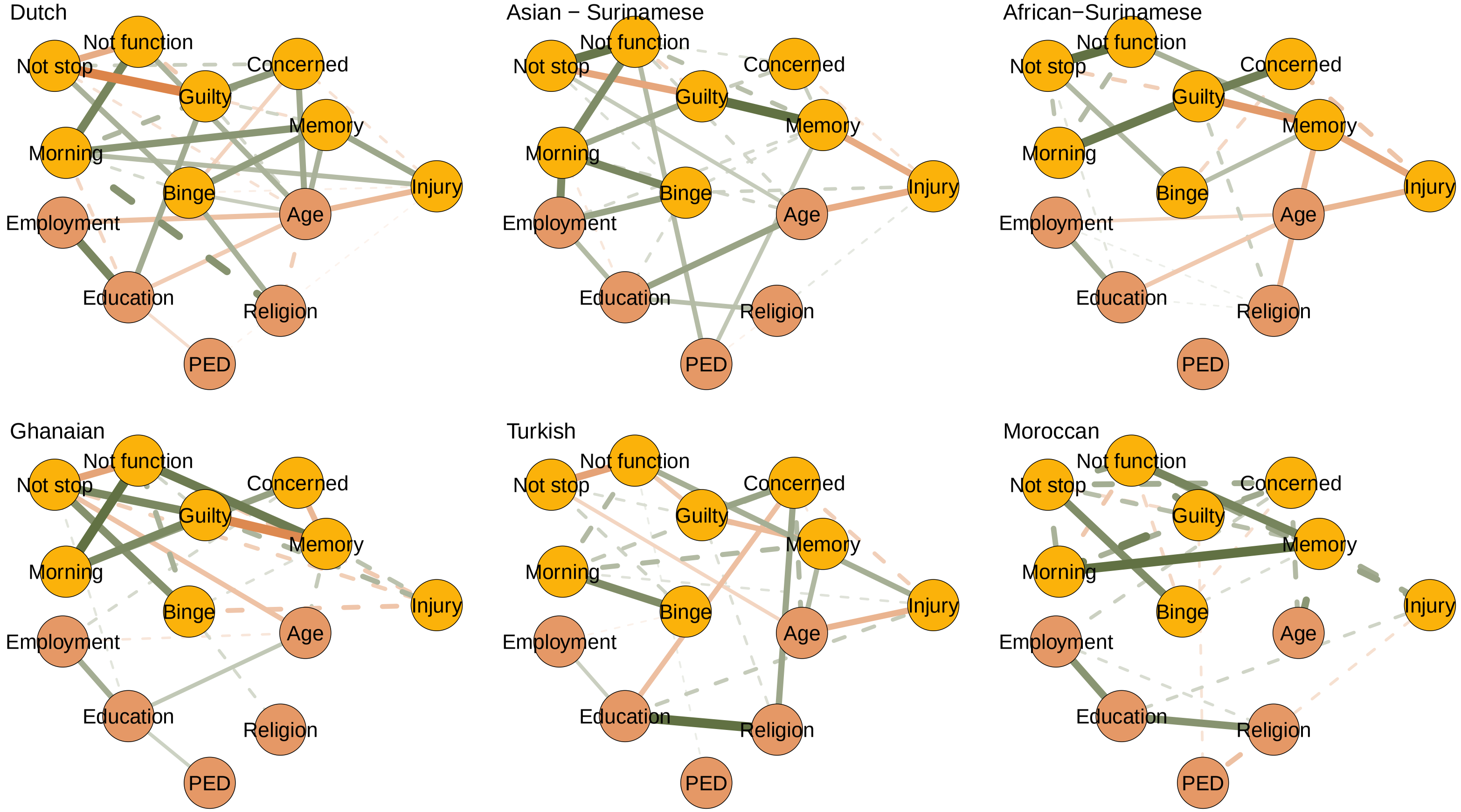

Note. Networks depicting the partial associations between AUD symptoms and sociodemographic determinants split by ethnic group for both sexes. Edge thickness and saturation represent the strength of association, the thicker the edge, the stronger the association. Red edges indicate negative relations and blue edges, positive ones. *Nodes:* Binge–binge drinking, Not stop–unable to stop drinking, Not function–unable to do normal activities, Morning–need a drink in the morning, Guilty–felt guilty due to drinking, Memory–unable to retrieve last night, Injury–injury due to drinking, Concerned–others concerned, PED–perceived ethnic discrimination.

Figure S7

Edge evidence plots for both sexes of the various ethnic subgroups.

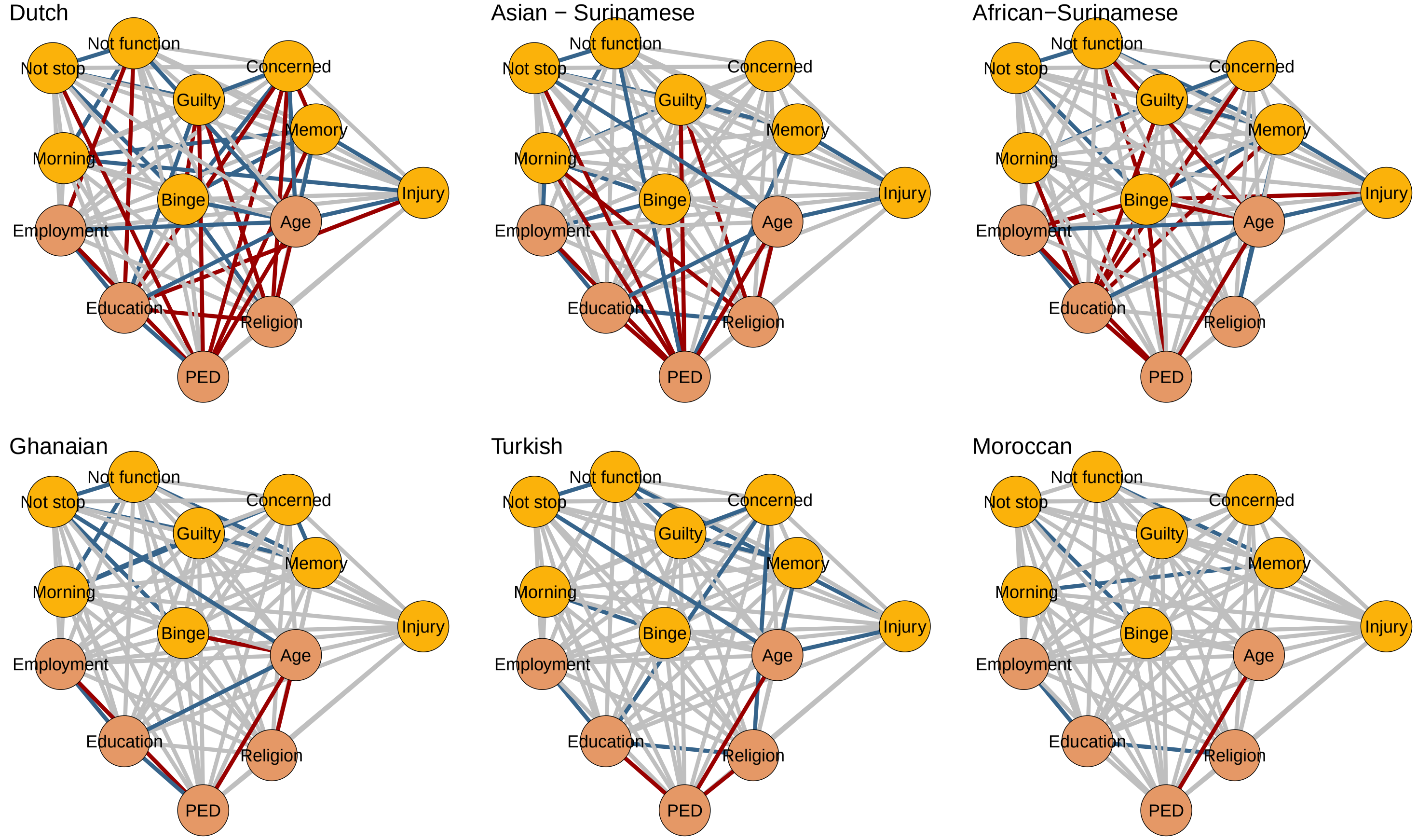

Note. Networks depicting the respective network uncertainty for both sexes in the ethnic subgroups. In the edge evidence plots, edges represent evidence for inclusion, where blue edges represent evidence for inclusion ($BF_{inc} > 10$), red edges evidence for exclusion ($BF_{inc} < 0.1$), and grey edges absence of evidence for either in- or exclusion ($0.1 < BF_{inc} < 10$). *Nodes:* Binge–binge drinking, Not stop–unable to stop drinking, Not function–unable to do normal activities, Morning–need a drink in the morning, Guilty–felt guilty due to drinking, Memory–unable to retrieve last night, Injury–injury due to drinking, Concerned–others concerned, PED–perceived ethnic discrimination.
